# The formulation of irrigation and nitrogen application strategies under multi-dimensional soil fertility targets based on preference neural network

**DOI:** 10.1038/s41598-022-25133-1

**Published:** 2022-12-03

**Authors:** Shuai Lou, Rui-Qi Hu, Yue Liu, Wan-feng Zhang, Shu-Qing Yang

**Affiliations:** 1 Water Conservancy and Civil Engineering College, Inner Mongolia Agricultural University, Hohhot, Inner Mongolia 010018 People’s Republic of China; 2 Lifin Tech., Co., Ltd., Beijing, 200433 People’s Republic of China; 3 Tourism College, Inner Mongolia Normal University, Hohhot, Inner Mongolia 010028 People’s Republic of China

**Keywords:** Environmental sciences, Computer science, Agroecology

## Abstract

With the aim of improving soil fertility, it is of great significance to put forward optimal irrigation and nitrogen fertilizer application strategies for improving land productivity and alleviating non-point source pollution effects. To overcome this task, a 6-hidden layer neural network with a preference mechanism, namely Preference Neural network (PNN), has been developed in this study based on the field data from 2018 to 2020. PNN takes soil total nitrogen, organic matter, total salt, pH, irrigation time and target soil depth as input, and irrigation amount and nitrogen application rate (N rate) as output, and the prior preference matrix was used to adjust the learning of weight matrix of each layer. The outcomes indicated that the predictive accuracy of PNN for irrigation amount were (R^2^ = 0.913, MAE = 0.018, RMSE = 0.022), and for N rate were (R^2^ = 0.943, MAE = 0.009, RMSE = 0.011). The R^2^ predicted by PNN at the irrigation amount and N rate were 40.03% to more than 99% and 40.33% to more than 99% higher than those obtained using support vector regression (SVR), linear regression (LR), logistic regression (LOR) and traditional back propagation neural network (BPNN), respectively. In addition, compared with the neural network (Reverse Multilayer Perceptron, RMLP) with the same structure but no preference structure, the R^2^ of the predicted irrigation amount and N rate by PNN increased by 25.81% and 27.99%, respectively. The results showed that, through the irrigation of 93 to 102, 92 to 98 and 92 to 98 mm, along with nitrogen applications of 65 to 71, 64 to 73 and 72 to 81 kg/hm^2^ at 17, 59 and 87 days after sowing, respectively, the organic matter, total nitrogen, total salt content and pH of the soil would reach high fertility levels simultaneously.

## Introduction

The development of agriculture in China largely depends on the input of chemical fertilizers^1^. However, excessive irrigation and nitrogen applications not only lead to low fertilizer utilization efficiency but also result in soil acidification^2^, soil nitrogen leaching losses^3^, decreased organic matter content levels^4^, and secondary salinization^5^, which ultimately result in low soil fertility. It has been observed that methods involving the deep burial of straw were able to alleviate nitrogen loss^6^ and soil salinization^7^, and also could increase the soil carbon storage levels^8^. However, a reasonable water and nitrogen application scheme must be taken to promote straw decomposition and improve soil fertility, reduce non-point source pollution effects, and realize salinization control. There have been few studies performed regarding the formulation of irrigation and nitrogen application strategies based on multi-indicator fertility goals under the condition of straw burial.

Over the past two decades, the majority of the accepted irrigation or nitrogen application strategies have only been able to formulate through comparisons of limited field treatments^9–12^. To reduce the costs of field experiments and improve the accuracy of irrigation and nitrogen application strategies, physical models such as HYDRUS^13,14^ and SWAP^15,16^ came into being. These types of models can simulate the effects of different irrigation schemes on soil solute under specific boundary conditions. The model’s prediction accuracy can be improved by increasing the input density of irrigation and nitrogen application^17^. However, most irrigation or nitrogen application optimization strategies proposed through physical models were based on the forward research concept of “treatment-index-treatment.” In other words, first, determine the influence trend of measure variables such as irrigation amount on farmland variables such as soil salinity, then deduce the optimal measure according to the trend. However, the accuracy of the strategies formulated under the aforementioned concept depends mainly on the range and density of the measure variables in the adopted field layouts or input models. In addition, the effectiveness of the irrigation or nitrogen application combinations that had not been included in the model input ranges could not be fully validated. On the other hand, the research approach based on “treatment-index-treatment” cannot build a direct mapping relationship between the input indicators and final results, and must conduct an additional screening layer. As a result, the model can only optimize the strategy for a single goal, and cannot consider multi-dimensional goals comprehensively. Therefore, in order to address those issues, an innovative research idea which could directly put forward irrigation and nitrogen fertilizer application optimization strategies according to determined targets was urgently required.

In recent years, artificial intelligence (AI) models have been successfully used in the prediction processes of farmland indexes, such as yields^18,19^, irrigation, nitrogen applications^20^. Some studies have successfully predicted citrus fruit numbers and coffee yields using SVR models trained by crop images^21,22^. In addition, wheat yields have been successfully predicted using high-resolution data collected by satellite sensors. The accurate predictions of soil total nitrogen, organic carbon, and water content have been achieved using SVR models trained by near-infrared spectral data^23^. Furthermore, the yields^24^ of crop ET_0_^25^ and oilseed rape have been accurately predicted using multi-layer perceptron (MLP) methods. In addition to successful predictions on time scales, Dong et al.^26^ used wavelet BP-neural networks to predict maize crop yields under various fertilization treatment conditions and established the mapping relationships between fertilization treatments and crop yields. Gu et al.^27^ used BP neural networks to predict crop yields under different amounts of irrigation. Therefore, it can be seen that the combinations of artificial intelligence and field experimental processes have provided new ideas for the formulation of irrigation and nitrogen application strategies. However, it should be noted that the models mentioned above had been mainly used for predicting such indicators as crop yields and had rarely shown the ability to be relevant in the formulation of accurate irrigation and nitrogen application strategies. In addition, the simple structure of the BP neural network leads to the lack of generalization ability, which cannot effectively solve the over-fitting problem caused by the limited amount of experimental data.

## Objectives

**Figure 1:**
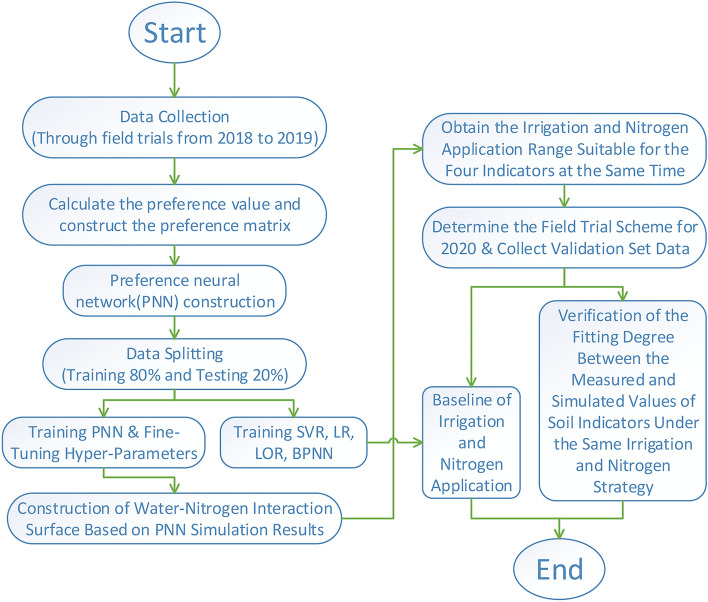
Flow chart of the proposed methodology to forecast evaporation using machine learning models.

The contribution of this study is to developed a preference neural network (PNN). The reverse training mechanism of PNN enables the model to directly formulate optimal irrigation and nitrogen application strategies based on multi-dimensional goals composed of soil organic matter, total nitrogen, total salt, and pH value. In the HeTao irrigated area of northwest China, we carried out a two-factor cross experiment in 2018 and 2019 on the amount of irrigation and nitrogen application. We trained the PNN model using the obtained data, and the irrigation-nitrogen interaction surface of a single fertility index was drawn according to the predicted results. The strategies of irrigation and nitrogen application that could simultaneously meet the four fertility targets of soil organic matter, total nitrogen, total salt and pH were obtained. Based on this strategy, we rearranged the experiment in 2020, and the feasibility of the irrigation-nitrogen application scheme was verified by evaluating the fitting degree of simulated and measured values under the same strategy. In addition, the performance of PNN was compared with Support Vector Regression (including linear, Poly, and RBF kernel functions), Linear Regression (LR), Logistic Regression (LOR), and traditional Back Propagation Neural Networks (BPNN) , and traditional BP neural network to verify the model performance. The training and performance evaluation processes is shown in Fig. 1.

## Materials and methods

### Study area

**Figure 2:**
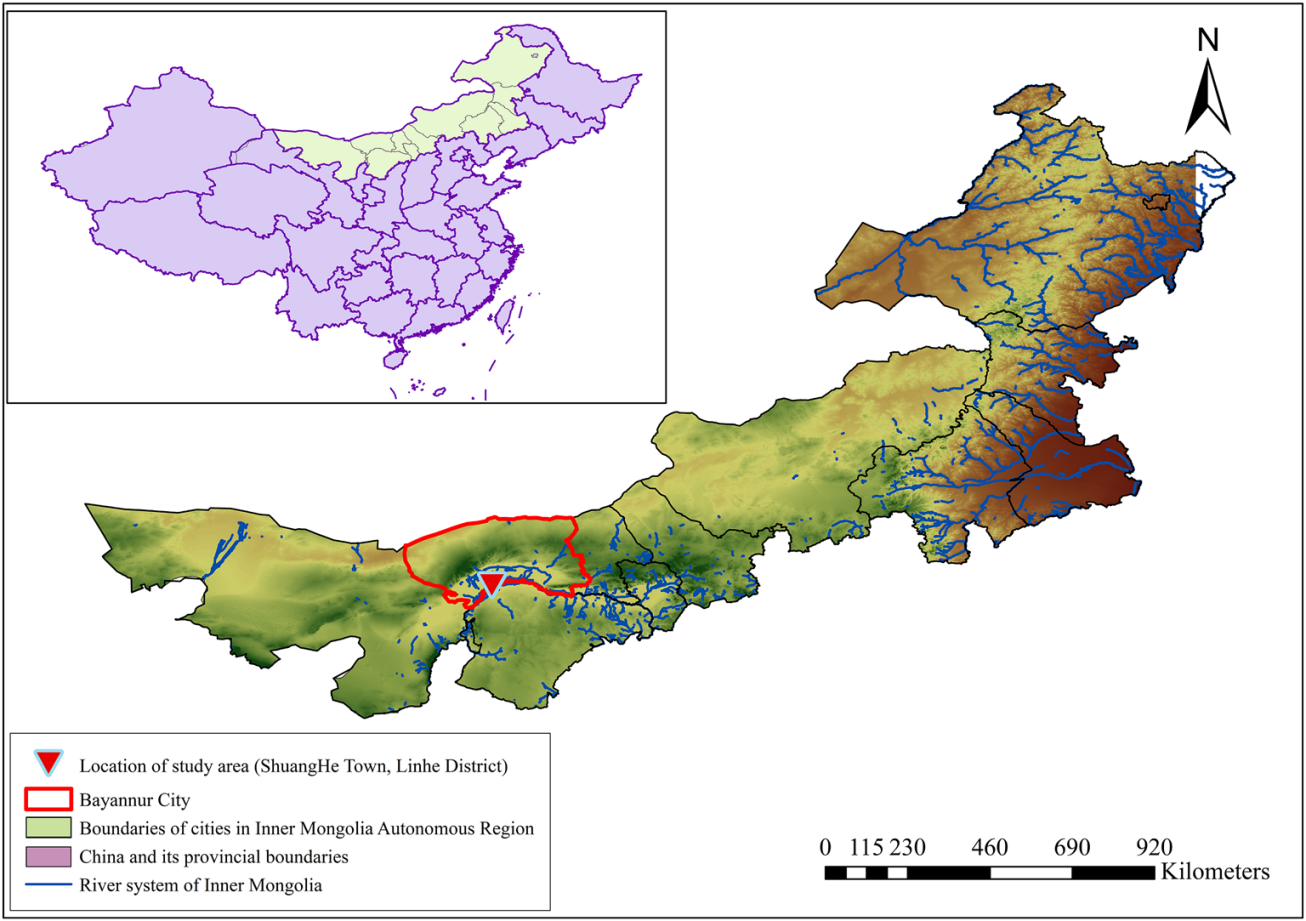
The location of the study area.

Figure 2 shows the location of the study area on a map of China generated by ArcGIS software. This study’s field experiments were carried out in the Shuanghe Town agricultural comprehensive water-saving demonstration area (40°42′ N; 107°24′ E), which is located in the middle reaches of the Hetao Irrigation Area of Inner Mongolia. The duration of the experimental process ranged from April in 2018 to October in 2020. The experimental area was characterized by a mid-temperate semi-arid continental climate. The average annual precipitation was determined to be 138 mm and the average evaporation was approximately 2332 mm. The majority of the rainfall was concentrated during summer and autumn seasons, and the accumulation of salt in the surface soil was considered to be serious in the spring and winter months. The average rainfall during maize growth period was 75.3 mm. The 0 to 40 cm soil layers in the experimental area were categorized as silty loam soil, with an average bulk density ranging from 1.42 to 1.53 g cm^−3^. A maize straw layer with a thickness of 5 cm was buried at a depth of 40 cm, and then the land was leveled. Also, in addition to autumn watering and spring irrigation procedures, water from the Yellow River was used three times for irrigation during the entire growth period of the maize crops. The adopted irrigation method belonged to border irrigation. Urea (46% N) were used as the fertilizer types.

### Field trials design and data collection

We carried out experiment 1 from 2018 to 2019, and the data obtained were used for model training and to determine the hyper-parameters. The experimental design is shown in Table 1. The PNN model trained from the data obtained in experiment 1 predicted the optimal range of irrigation amount and nitrogen application rate (N rate) for each growth period of maize. In these ranges, the soil organic matter and total nitrogen could be kept above 20 g/kg and 1.6 g/kg, respectively, the soil salt content was less than 2 g/kg, and the pH value was between 6.5 and 7.5. In order to verify the accuracy and feasibility of the range of irrigation and nitrogen application simulated by PNN, the field experiment 2 was set in 2020 based on the range simulated by PNN and to evaluate the fitting degree between measured and simulated values of soil indicators under the same amount of irrigation and nitrogen application. The experimental design is shown in Table 2.

**Table 1:** Experimental 1 design scheme.

Serial number	Treatment	Irrigating water quota (mm)	Nitrogen application rate (kg/hm^2^)
1	W_1_N_1_	60	45
2	W_1_N_2_	60	60
3	W_1_N_3_	60	75
4	W_1_N_4_	60	93.3
5	W_2_N_1_	90	45
6	W_2_N_2_	90	60
7	W_2_N_3_	90	75
8	W_2_N_4_	90	93.3
9	W_3_N_1_	120	45
10	W_3_N_2_	120	60
11	W_3_N_3_	120	75
12	W_3_N_4_	120	93.3

**Table 2:** Experimental 2 design scheme.

Treatment	17d		59d		87d	
	Irrigating water quota (mm)	Nitrogen application rate (kg/hm^2^)	Irrigating water quota (mm)	Nitrogen application rate (kg/hm^2^)	Irrigating water quota (mm)	Nitrogen application rate (kg/hm^2^)
W_1_N_1_	102	71	98	73	98	81
W_1_N_2_	102	71	98	73	98	81
W_1_N_3_	102	71	98	73	98	81
W_2_N_1_	97.5	68	95	68.5	95	76.5
W_2_N_2_	97.5	68	95	68.5	95	76.5
W_2_N_3_	97.5	68	95	68.5	95	76.5
W_3_N_1_	93	65	92	64	92	72
W_3_N_2_	93	65	92	64	92	72
W_3_N_3_	93	65	92	64	92	72

The experimental design were repeated for three times. The plot area of each treatment measuring 8 × 9 = 72 m^2^. The surrounding area was separated using 1.2 m buried polyethylene plastic film, and 30 cm was left at the top to prevent fertilizer and water from flowing into each other. The field management process was consistent with that used by the local farmers. The film width of maize was 1.1 m, with each film covering two rows. The plant spacing was approximately 45 cm, and the row spacing was 35 cm. In addition, the planting density of the maize was 60,000 plants/hm^2^.

During the entire growth period of the maize crops, soil samples were collected from the 0 to 20 cm, 20 to 40 cm, 40 to 60 cm, 60 to 80 cm, and 80 to 100 cm soil layers using a soil drill and a three-point method was adopted. The soil samples were stored at 4 °C for the determination of total nitrogen, organic matter, total salt content, and pH values. The total nitrogen, organic matter, total salt content, and pH were determined using a KDN-AA double tube azotometer, MWD-2 microwave universal digestion device, TU1810PC ultraviolet–visible spectrophotometer, and a TU18950 double beam ultraviolet–visible spectrophotometer, respectively.

Soil parameters measured include organic matter (SOM), total nitrogen (TN), Salt and pH. The data set includes pre-irrigation and post-irrigation reports from 2018 to 2020. Statistical parameters regarding the soil data are shown in Table 3.

**Table 3:** Various meteorological variables and their descriptive statistics.

Dataset	Xmean	Xmedian	Xmin	Xmax	SD
SOM (g/kg)	15.45	15.36	4.27	21.79	2.93
TN (g/kg)	1.43	1.44	1.02	1.72	0.12
Salt (g/kg)	1.43	1.35	0.74	2.85	0.46
pH	7.49	7.42	6.71	8.45	0.36

In this table, the Xmean, Xmedian, Xmax, Xmin and SD represent the mean, median, maximum and minimum of the weather variables, standard deviation, respectively.

The dataset obtained in Experiment 1 in 2018 to 2019 was 2490 rows in size, the 80/20 principle was used to data into training, and testing sets were required for ML modeling; 80% of data were employed for model training, while the remaining 20% were used for testing. Specifically, the data corresponding to the treatments with the nitrogen application rate (N rate) of 75 kg/hm^2^ (N3) in all the treatments (W1N3, W2N3, W3N3) were used as the test set, and the data of the other treatments were used as the training set. The training set was used to initiate ML parameter training. Subsequently, The test set was employed to assess the model. The dataset size in 2020 was 1080 rows, which was used to verify ML modeling.

**Figure 3:**
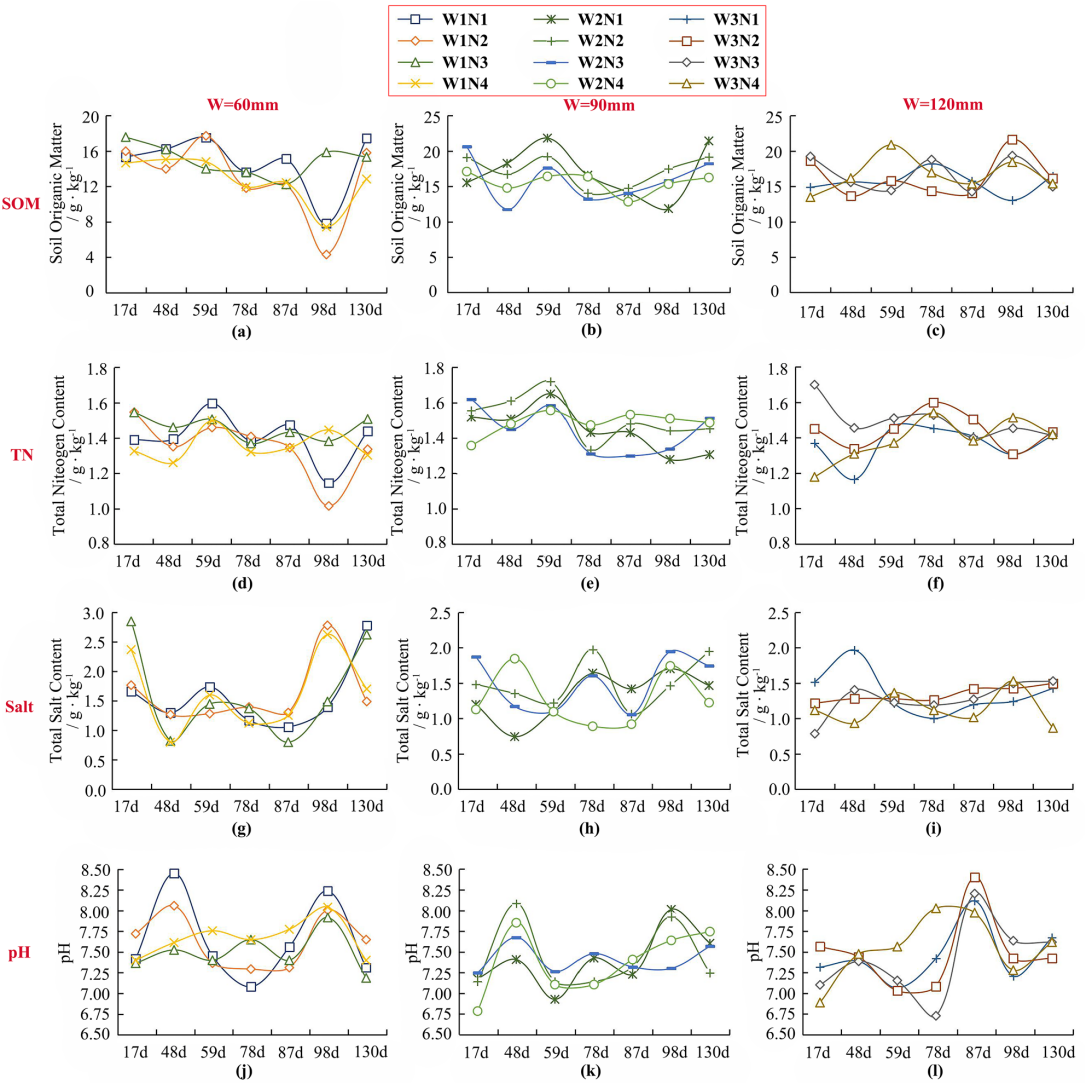
Changes in soil organic matter, total nitrogen, salinity, and pH under different treatments over time (a case study of 2019).

Figure 3 shows the changes of soil indexes over time for each treatment in the field test (take the 0–40 cm soil in the main distribution area of maize roots as an example). There are differences under the influence of different irrigation amounts. When irrigation is 90 mm, soil SOM is 13.25% and 7.00% higher than 60 mm and 120 mm, and soil TN is 4.59% and 6.50% higher than 60 mm and 120 mm, respectively. The soil Salt was 23.30% lower than 60 mm, and the pH was 4.16% and 4.36% lower than that of 60 mm and 120 mm, respectively. It can be seen that irrigation of 90 mm is more favorable for increasing soil SOM and TN contents and reducing soil salinity and alkalinity. Soil SOM and TN contents were the highest at n 75 kg/hm^2^, which were 4.38% and 8.34% higher than those at N = 93.3 kg/hm^2^, respectively. Soil Salt was the lowest at N = 60 kg/hm^2^, which was 3.02% lower than those at N = 75 kg/hm^2^, with a small gap with other levels. In conclusion, nitrogen application of 75 kg/hm^2^ was beneficial to increase soil organic matter and nitrogen content, and nitrogen application of 60 kg/hm^2^ was beneficial to controlling soil salt content.

### Machine learning (ML) models used for irrigation and nitrogen application strategies

Five ML frames were used to estimate the irrigation and N rate. These models are preference Neural Network (PNN), Support Vector Regression (SVR), Linear Regression (LR), Logistic Regression (LOR), and traditional BP Neural Networks (BPNN). Among them, the prediction effects of linear, Poly, and rbf kernel functions are respectively tried in SVR framework. The torch framework was used to train and test machine learning models in Python.

### Development of preference neural network

#### Model framework

The preference neural network (PNN) which was proposed for the first time in this study was a typical deep learning model. PNN can be regarded as an approximate natural function in order to describe the complete dependence of the soil fertility indexes, including the effects of soil total nitrogen, organic matter, total salt content, and pH values on irrigation and nitrogen applications. More specifically, PNN has the ability to optimize the function by constructing the mapping y = f (x, θ) and learning parameter θ.

**Figure 4:**
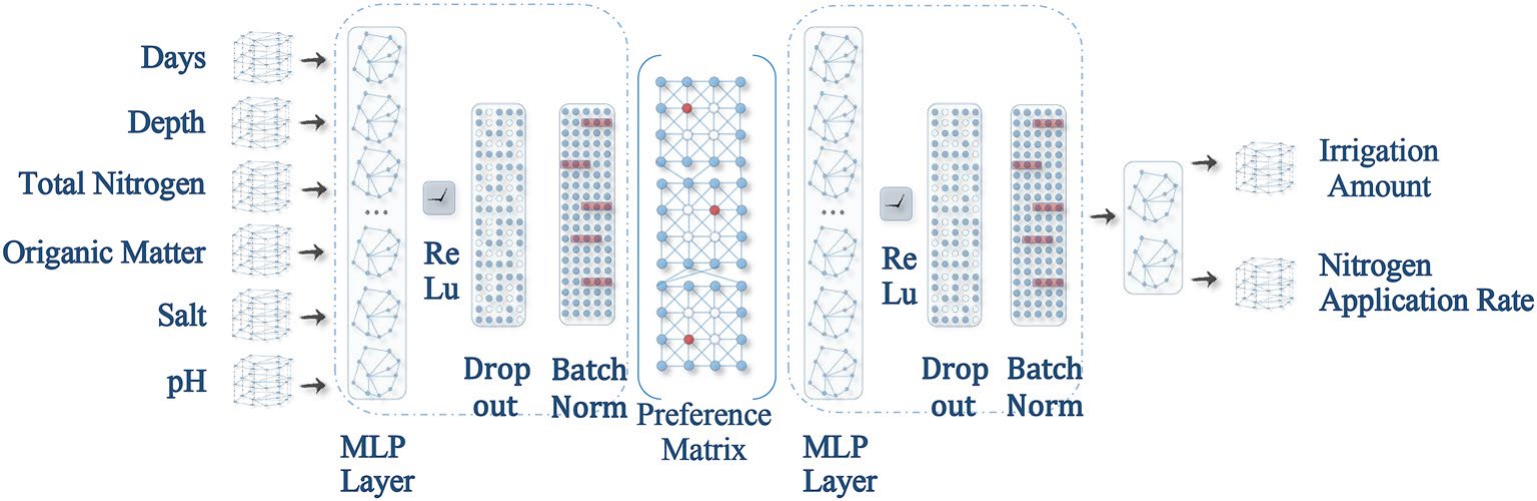
Schematic diagram for the PNN structural connections. In the figure, it can be seen that when each input vector passed through each layer of the PNN, it is first multiplied by the Hadamard product of the weight matrix and preference value matrix for the purpose of obtaining a weight matrix with preference properties. After the matrix was activated by the Relu Function, Batch Normalization Module Methods and the Dropout Module were used for random suspension and normalization processing, and the input of the next layer was obtained.

First, the input end of PNN model was defined as matrix *X* ∈ ℝ^*n*×*d*^ (in which *n* is the sample size, *n* = 2490; and *d* is the dimension of each input vector, *d* = 6), where {*x*_*i*_} _*i*=*1, …, n*_ ∈ *X* represents the vectorized set of total nitrogen, organic matter, salt content, and pH used for measuring the soil fertility, as well as the nitrogen application and irrigation durations (expressed by days after sowing). At the same time, the output end of the model was defined as the matrix *Y* ∈ *ℝ*^*n*×2^, which represented the levels of the irrigation and nitrogen fertilizer applications. The goal of the proposed PNN model was to learn the fixed mapping *Y*′ = *f* (*X*; *θ*) **⇒***Y* through the given input matrix *X*, where *θ* is the well optimized learnable parameters which can be obtained via PNN training. Meanwhile, the predicted value *Y′* will infinitely approach the measured value *Y*. The structure and the algorithm of this study’s PNN model is shown in Fig. 4 and Table. 4.

**Table 4:**
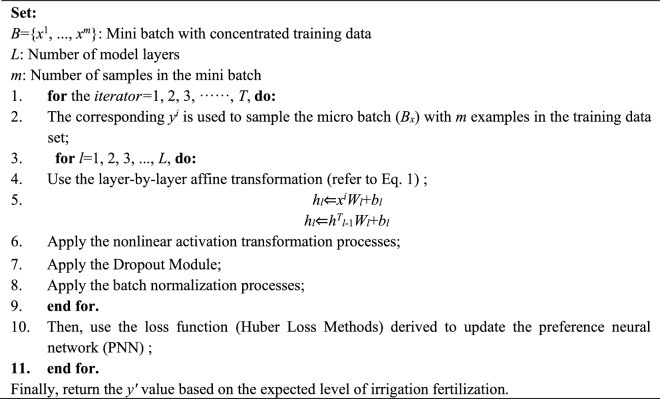
Algorithm of Preference neural network.

#### Layer-by-layer affine transformation

A good definition of the affine transformation of the information flow between layers is considered to be the key to neural network model training. Generally speaking, the learnable parameter θ of each layer of a model includes the weight parameter w and the preference parameter b. The hidden representation h_l_ of the l-th layer in PNN is defined as follows:

1 $${h}_{l}({h}_{l-1};{W}_{l},{b}_{l})={h}_{l-1}^{\mathrm{T}}{W}_{l}+{b}_{l}$$

where *W*_*l*_ and *b*_*l*_ represent the learnable weight and bias variables of the *l* layer, respectively, and *h*_*l*-1_ is the hidden representation of the upper layer. Therefore, when *l* = 1, then *h*_0_ = *X*.

In the present study, using the hierarchical update rules, a given input data stream was allowed to pass through each hidden layer with intermediate operations, and then finally reached the output end.

#### Preference structure

The correlation between different production behavior factors (e.g., irrigation levels) and different natural factors (e.g., soil organic matter) differs in agricultural production. However, the traditional fully connected neural network has the characteristic that nodes of one layer are fully connected with all nodes of subsequent layers, resulting in the neurons between production behavior factors and natural factors with very weak correlation still all being connected. Conversely, connections between neurons corresponding to factors with solid correlations are not strengthened.

Therefore, in this study the preference value module was specially developed. By first calculating the correlation and significance between different production behavior factors (irrigation amount, N rate) and different soil fertility factors (organic matter, total nitrogen, total salt and pH), the preference value between the above two types of variables was calculated, and the preference matrix was constructed. Then the Hadamard product of the weight matrix and preference matrix was used to realize the artificial intervention and guidance to the neural network’s learning process.

In order to reduce the adverse impact of non-normality of data on correlation analysis as much as possible, this study rank-based inverse normal (RIN) transformations (i.e., conversion to rank score) methods were used to normally process the data^28^. The RIN transformation function used here is as follows:

2 $$f(x)={\Phi }^{-1}\left(\frac{{x}_{r}-\frac{1}{2}}{n}\right)$$

where *Φ*^*–1*^ is the inverse normal cumulative distribution function, and *n* is the sample size.

The normal cumulative distribution function is represented as follows: for discrete variables, the sum of probabilities of all values less than or equal to a, and its formula is as shown below:

3 $${F}_{X}(a)=P(X\le a)$$

The RIN normalized conversion values meet the requirements of normal distribution, Pearson correlation analysis and t-test can be directly performed, and the formula used was as follows:

4 $$r(X,Y)=\frac{\mathrm{Cov}(X,Y)}{\sqrt{\left(\mathrm{Var}\left[X\right]\mathrm{Var}\left[\mathrm{Y}\right]\right)}}$$

where *r* (*X, Y*) is the Pearson Correlation Coefficient, *Var* [*X*] is the variance of *X,* and *Var* [*Y*] is the variance of *Y*, *Cov* (*X, Y*) is the covariance of *X* and *Y*, which represents the overall error of the two variables. The t-test is performed on the normalized data after rank-based inverse normal (RIN) transformation method, and the formula is as follows:

5 $$t=\sqrt{\frac{n-2}{1-{r}^{2}}}$$

where *n* is the number of samples, and *r* represents the Pearson Correlation Coefficient. Preference value is the concentrated embodiment of correlation and significance between variables, and the calculation formula is as follows:

6 $${PV}_{ij}=\frac{r({X}_{i},{Y}_{j})}{{P}_{ij}+e}$$

where *PV*_*ij*_ represents the preference values between the variables *X*_*i*_ and *Y*_*j*_, *X*_*i*_ represents the *i*th production behavior factor (e.g., irrigation amount), and *Y*_*j*_ represents the *j*th soil fertility factor (e.g., soil organic matter content), ${P}_{ij}$ is obtained by looking up the table based on the *t*, and *e* is a constant, taking 0.001 in order to prevent the denominator of the formula from being 0.

In order to make the preference values of the various indicators in the same order of magnitude more stable, the preference values were normalized:

7 $${PV}_{normal}=\pm \frac{\left|{PV}_{i}-{PV}_{avg}\right|}{\sqrt{\frac{\sum_{i=1}^{N}{({PV}_{i}-{PV}_{avg})}^{2}}{N-1}}}$$

where *N* represents the number of variables related to the experimental treatments, *PV*_*i*_ -*PV*_*avg*_ takes the absolute value, while the positive or negative values of the *PV*_*normal*_ were determined by the positive or negative values of the correlation *r*.

**Figure 5:**
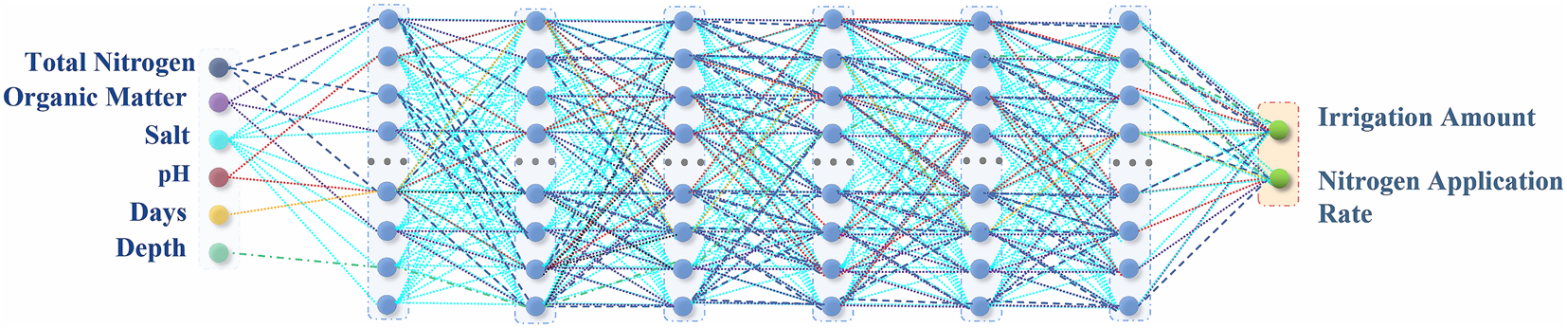
Schematic diagram of the preference connection structures of the preference neural networks. The depth of the network detailed in the figure only illustrates the preference connection structure (for a better demonstration), and does not indicate the depth of the PNN used in the experiment.

**Figure 6:**
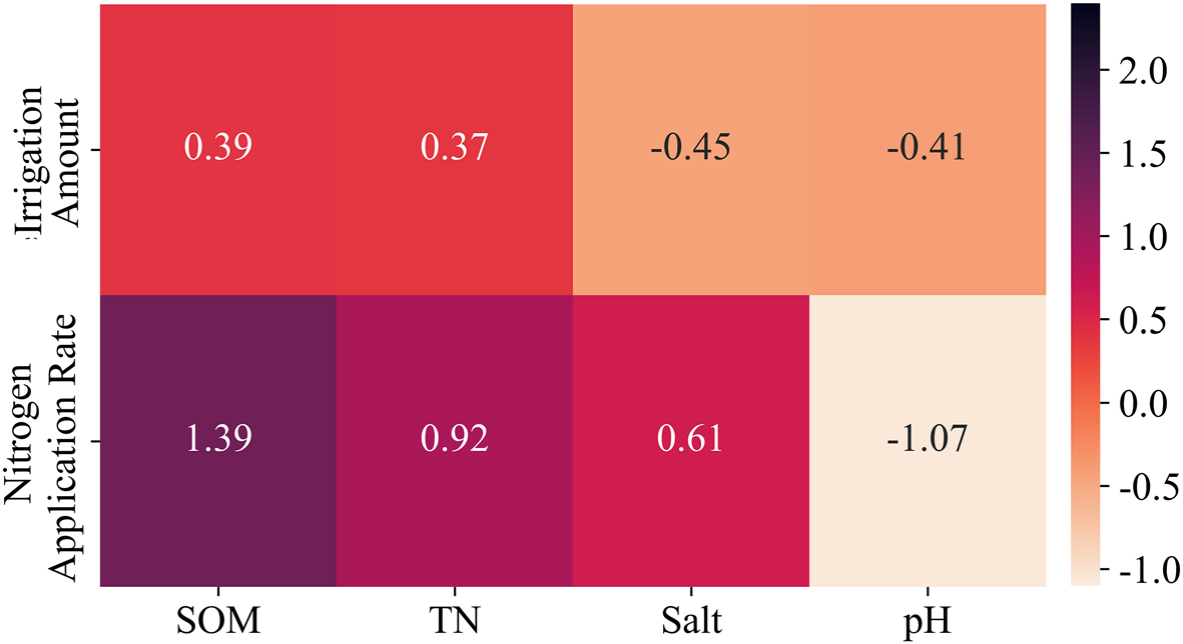
*PV*_*normal*_ between production behavior factors and natural factors. Since soil depth, days, irrigation amount and N rate were all artificially set variables, and there was no objective correlation in the data set. Therefore, the preference values among these variables were default *e* = 0.001.

The PNN integrated the preference matrixes into the neural network structures by identifying the Hadamard products of the learnable weights between the preference matrixes and the input and output data. By referring to Eq. (1) in the hierarchical affine transformation, the preference constraint of PNN could be expressed as follows:

8 $${h}_{l}({h}_{l-1};{W}_{l},{b}_{l})={h}_{l-1}^{T}{W}_{l}\odot P+{b}_{l}$$

where *P* is the preference matrix calculated by Eq. (8), and ⊙ represents the Hadamard product of the corresponding elements of the matrix. The structure of preference neural network and preference value are shown in Figs. 5 and 6.

#### Hyper-parameters of PNN

We conducted experiments on the datasets with varying the hyper-parameters (such as the number of PNN layers and hidden layers, the number of nodes in each layer, learning rate, dropout rate and batch size) to understand that how the Hyper-parameters impact on the performance of PNN.

We select the activation function and learning rate by referring to the neural network structure commonly used in similar fields (1 hidden layer and 64 hidden nodes)^29,30^. It is found that ReLU has better performance than other activation functions (sigmoid, tanh). The performance is best when the learning rate is around 0.005. It is generally believed that neural networks with more hidden layers are able, with the same number of resources, to address more complex problems^31^, but excessively increasing network depth will easily lead to overfitting^32^. Since there is no direct method to select the optimal number of hidden layers and nodes^33^, this study first calculated the structure of one hidden layer and 64 nodes in each layer, and found that the combined effect was poor (R^2^ of irrigation and nitrogen application were 0.3971 and 0.4124, respectively). Therefore, the trial-and-error method is adopted. The number of hidden layers starts from 1 and is incremented by 1 to test the maximum number of 10 hidden layers. The number of nodes in each layer were tested with a maximum number of 100 hidden neurons, starting with 5 and increasing by 5.

We found that when the number of hidden layers of PNN exceeds 6, and the number of nodes in each layer exceeds 65, the performance will drop significantly. The reason behind this phenomenon could be the current dataset size is insufficient for larger scale of the PNN model. In the consideration of that the size of new dataset we can obtain very year is similar to the current dataset size, we believe that current hyper-paramter settings of PNN is in a reasonable condition.

After that, the number of layers was fixed as 6, and the number of nodes in each layer were tested 10 times with 60 as the starting point and 1 as the increment, we found that when the number of nodes was 64, the improvement of the fit degree was no longer noticeable. On this basis, we changed different activation functions and learning rate again, and found that PNN still has the best performance when the activation function is ReLU and the learning rate is 0.005. Then, different batch sizes and dropout rates were tried. The two parameters had weaker effects on the performance than the other parameters, and the performance was optimal at 256 and 0.1, respectively.

The hyper-parameters include:

1. number of PNN layers;
2. number of hidden layers;
3. types of activation function;
4. percentage of dropout;
5. learning rate;
6. loss function;
7. optimizer;
8. batch size;
9. number of epochs;
10. number of workers.

The ideal PNN structure for the study comprises these layers:

1. number of PNN layers is 8;
2. number of hidden layers is 6;
3. Fully connected layers with 64 nodes and ReLU activation function
4. dropout with 0.1.
5. the learning rate is 0.005;
6. loss function is Huber Loss Methods (HLM);
7. optimizer: ADAM;
8. epochs is 500;
9. the batch size is 256;
10. number of workers is 6.

### Hyper-parameters of other models

LR algorithms and LOR do not have hyper-parameters that need to be adjusted. A part of the hyper-parameters of the SVR model was determined by referring to Guan Xiaoyan's research^34^, and a part of the hyper-parameters of the BPNN model was determined by referring to Gu Jian's research^27^. RMLP takes the same hyperparameters as PNN. The hyperparameters of SVR and BPNN models are shown in Table 5.

**Table 5:** Hyper-parameters of other model.

SVR	BPNN	RMLP
1. The penalty value C on the samples out of error ε is 26 2. *ε* is used to control the size of the regression approximation error pipeline, and its value is 0.036	1. Number of PNN layers is 3 2. Number of hidden layers is 1 3. Number of hidden layer nodes is 13 4. The learning rate is 0.05 5. The maximum number of training times to 10,000	1. Number of PNN layers is 8 2. Number of hidden layers is 6 3. Number of hidden layer nodes is 64 4. The learning rate is 0.005 5. The maximum number of training times to 10,000

### Model performance evaluation

The proposed PNN model was trained and validated using the field measured data from 2020 and the performance achievements of PNN were evaluated by the root mean square errors, mean square errors, and mean absolute errors as follows:

9 $$RMSE=\sqrt{\frac{{\sum }_{i=1}^{n}{({y}_{ipre}-{y}_{imea})}^{2}}{n}}$$

10 $${R}^{2}=1-\frac{{\sum }_{i=1}^{n}{({y}_{ipre}-{y}_{imea})}^{2}}{{\sum }_{i=1}^{n}{({y}_{ipre}-{y}_{iavg})}^{2}}$$

11 $$MAE=\frac{{\sum }_{i=1}^{n}\left|{y}_{ipre}-{y}_{iavg}\right|}{n}$$

### Model multidimensional fertility targets

The soil fertility grade classification of soil organic matter, soil total nitrogen content and salt content in this study was based on the soil fertility grade classification results by the Agriculture and Animal Husbandry Bureau of Bayannur City, along with the local standard Technical Specifications for the Assessment and Rating Criteria of Cultivated Land Quality (DB 15/T 1086, 2016), as the shown in Tables 6 and 7.

**Table 6:** Soil organic matter and Soil total nitrogen degrees.

Fertility degree	Extremely lack	Lack	Medium	High
Soil organic matter (g/kg)		< 10	10 to 20	> 20
Soil total nitrogen (g/kg)	< 0.41	0.41 to 0.87	0.87 to 1.60	> 1.60

**Table 7:** Grading of the salinization degrees.

Fertility degree	Non-saline	Light saline	Medium saline	Heavy saline	Saline
Concentration (g/kg)	< 2	2 to 4	4 to 6	6 to 10	> 10

In the evaluation system of soil fertility referencing the *Technical Specifications for Assessment and Rating Criteria of Cultivated Land Quality* (DB 15/T 1086, 2016), the pH was divided into four grades according to the membership degrees of the land productivity evaluations, as detailed in Table 8.

**Table 8:** pH grading degrees of the cultivated land.

Degree	1	2	3	4
pH	> 8.5	< 6.5	7.5 to 8.5	6.5 to 7.5

Based on the classification standard of soil fertility obtained by the Bureau of Agriculture and Animal Husbandry of Bayannur City, when the farmland soil is at the high fertility level, the soil organic matter and total nitrogen content should be more than 20 g/kg and 1.6 g/kg, respectively. Soil salt content was less than 2 g/kg. Meanwhile, the pH value is kept between 6.5 and 7.5.

## Results and discussion

### Construction of coupling simulation surface of irrigation and nitrogen application and feasibility verification of optimal irrigation-nitrogen application strategy

To reflect the corresponding changes in the water and fertilizer strategies caused by the variations in the single fertility indexes, During the simulation, fertilization and irrigation dates were set at 17, 59, and 87 days after sowing, respectively, which were consistent with the actual measures taken by local farmers, and the soil depths were taken as the mean value of the 0 to 40 cm maize root layers. More importantly, in the 4 variables (organic matter, total nitrogen, total salt, and pH), 3 of them were defined as fixed values, which were 20 g/kg, 1.6 g/kg, 2 g/kg, and 7.5 respectively. The range of changing value refers to the maximum and minimum values of the measured values in the 3-year experiment. The data set of the input model of this variable was obtained by very small steps, and a complete data set was formed with the fixed variables and input into the model.

**Figure 7:**
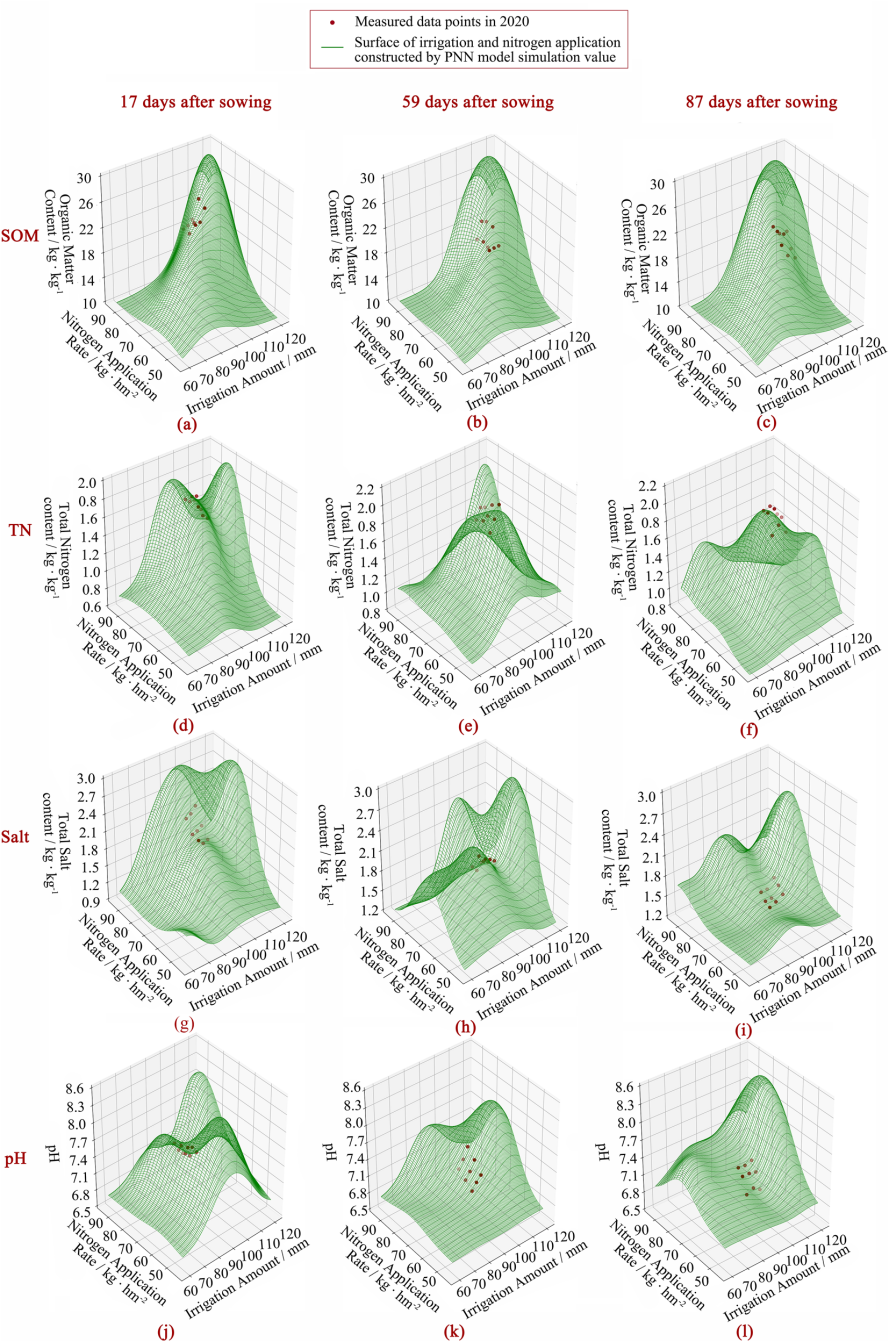
Surface of soil fertility index after irrigation and nitrogen application at seeding, jointing, filling stage of maize. The green surface is the water-nitrogen interaction surface with a single index change, which is obtained by PNN training based on the measured data from 2018 to 2019. The red dots are the measured values of corresponding indexes under the reset irrigation and nitrogen application levels in 2020.

In Fig. 7 and Table 9, taking 1/500 of the value range of each indicator as the step length, PNN was used to predict and verify the irrigation and nitrogen strategy under the change of a single indicator at each growth stage of maize, and Kriging interpolation was carried out. Meanwhile, the simulated values of 0 to 40 cm soil organic matter, total nitrogen, total salt, and pH were compared with those of PNN under the same water and nitrogen strategy in experiment 2 carried out in 2020 to verify the effectiveness of the PNN model.

**Table 9:** The comparison between the measured values of 0 to 40 cm soil indexes in each treatment obtained by the experiment in 2020 and the simulated values obtained by PNN under the same strategy.

	Soil fertility index	R^2^	MAE	RMSE
Seeding stage	SOM	0.7408	0.8472	0.9456
	TN	0.8235	0.0245	0.0263
	Salt	0.8405	0.0215	0.0252
	pH	0.9242	0.0857	0.0956
Jointing stage	SOM	0.7969	0.8120	0.8915
	TN	0.7605	0.0269	0.0299
	Salt	0.8105	0.0227	0.0271
	pH	0.9003	0.1058	0.1228
Filling stage	SOM	0.7358	0.8616	0.9556
	TN	0.9074	0.0180	0.0202
	Salt	0.9643	0.0305	0.0338
	pH	0.9147	0.0569	0.0676

As shown in Fig. 7a–c, it can be seen that with the increase of the target value of soil organic matter, the simulated amount of nitrogen application and irrigation showed an increasing trend. However, those trends had ceased when the soil organic matter reached 28 g/kg. As shown in Fig. 7d–f, soil total nitrogen content showed an overall increasing trend with the increase of N rate and presented a bimodal distribution with the increase of irrigation amount. When the N rate was 70 kg/hm^2^ and the irrigation amount was 95 mm, soil nitrogen reached the peak at the seeding stage, and total nitrogen content decreased with increasing irrigation and N rate. At the jointing stage, soil nitrogen reached the peak when the amount of irrigation and fertilizer was the maximum in the simulation range. At the filling stage, the total soil nitrogen began to decrease when the amount of nitrogen was more than 80 kg/hm^2^. As shown in Fig. 7g–i, soil salinity increased with the increase of irrigation and nitrogen application and reached the peak value when irrigation and nitrogen application reached 100 to 110 mm and 70 to 90 kg/hm^2^, respectively. Further increase in irrigation and nitrogen application would decrease the soil salinity level. As shown in Fig. 7j–l, soil pH showed a double peak trend with the increase of irrigation amount, reaching the first small peak at about 80 mm and then decreasing to the peak at about 120 mm. With the increase in N rate, soil pH showed single peak distribution at the seedling stage, jointing stage, and filling stage, and reached the peak at 77, 73 and 57 kg/hm^2^, respectively, and then decreased.

As shown in Table 9, under the same irrigation strategy, the fitting degree of corresponding soil TN, Salt, and pH in PNN simulation results was the highest with the measured values at the filling stage (R^2^ is 0.9074, 0.9643, and 0.9147, respectively). The fitting degree of corresponding SOM was the highest at the jointing stage (R^2^ = 0.7969). Among the four indicators, PNN has the highest fitting degree to pH, and R^2^ is above 0.9. Salt is the second, TN is the third, and its fitting degree to SOM is the lowest, R^2^ is between 0.7 and 0.8.

### Estimation of irrigation amount and nitrogen application rate in key growth period using preference neural network model

In experiment 2, soil organic matter, total nitrogen, salt, and pH of each layer within 0 to 100 cm of each treatment were taken as the target values. Input linear Support Vector Regression(Linear SVR), Poly Support Vector Regression(Poly SVR), RBF Support Vector Regression (rbf SVR), linear regression (LR), logistic regression (LOR), traditional BP Neural Networks (BPNN) were used to output the irrigation amount and N rate at 17d, 59d and 87d respectively. The simulation results were compared with the actual irrigation amount and N rate of each treatment in experiment 2 to verify the prediction accuracy of each machine learning model on irrigation amount and N rate. In order to investigate the effect of preference structures on improving the generalization performance of PNN, the reverse multilayer perceptron (RMLP), which has the same structure as PNN except without preference module, is also trained and output the same as other models.

The values pertaining to R^2^, MAE, RMSE are listed in Table 10. The model with the best performance is the Preference Neural Networks (The R^2^ of irrigation and nitrogen application were predicted to be 0.91, 0.94), followed by BPNN (R^2^ were 0.65 and 0.67). The R^2^ values predicted by the proposed PNN at the irrigation and nitrogen application were 40.03% to more than 99% and 40.33% to more than 99% higher than those obtained using the linear SVR, poly SVR, rbf SVR, LR, LOR, and traditional BP neural networks, respectively. Compared with RMLP without preference mechanism and with the same structure, R^2^ of PNN increased by 25.81% and 27.99%, respectively, when predicting irrigation amount and N rate. Compared with the conventional BPNN, the R^2^ of RMLP was increased by 10.14% and 8.69% when predicting irrigation amount and N rate, respectively. These results indicate that the preference structure can improve the generalization performance of the neural network for predicting irrigation volume and nitrogen application, and the performance of the neural network with 6 hidden layers and 64 nodes is better than that of the neural network with 1 hidden layer and 13 nodes. At the same time, compared with other machine learning models, the SVR model with poly and rbf as the kernel function obtained less accurate prediction of irrigation amount(R^2^ were 0.14 and 0.23) and more inaccurate prediction of N rate obtained by poly SVR (R^2^ was 0.27). Aside from the linear SVR model, the accuracy of the other SVR models was slightly lower than those of two regression models (LR and LOR) and two neural network models (PNN and traditional BP neural networks). This result may be explained by the fact that the prediction accuracy of SVR model is excessively sensitive to the influence of kernel function and hyper-parameter^35,36^.

**Table 10:** Model performance parameters.

	Model		R^2^	MAE	RMSE
Irrigation	SVR	Linear	0.5774	0.8292	0.9921
		Poly	0.1422	0.7525	0.9267
		rbf	0.2343	0.7148	0.8755
	LR		0.3732	0.8323	0.9689
	LOR		0.4902	0.8847	0.9408
	BPNN		0.6521	0.0834	0.0995
	RMLP		0.7257	0.0613	0.0756
	PNN		0.9130	0.0179	0.0216
Nitrogen application	SVR	Linear	0.6371	0.7837	0.9419
		Poly	0.2711	0.7016	0.8547
		rbf	0.4503	0.5683	0.7412
	LR		0.4406	0.879	0.996
	LOR		0.5305	0.8842	0.8568
	BPNN		0.6728	0.0591	0.0742
	RMLP		0.7369	0.0697	0.0835
	PNN		0.9432	0.0091	0.0114

### Formulation of irrigation and nitrogen application schemes based on multi-dimensional fertility targets

As shown in Table 11, Based on the surface of irrigation-nitrogen interaction constructed above, the intersection of irrigation amount and N rate was selected as the optimization strategy interval when all indexes were in the state of high fertility. When the irrigation amount was 93 to 102, 92 to 98, and 92 to 98 mm, and the N rate was 65 to 71, 64 to 73, and 72 to 81 kg/hm^2^ on the 17th, 59th and 87th days after sowing, respectively, the soil organic matter and total nitrogen could be kept above 20 g/kg and 1.6 g/kg, respectively, the soil salt content was less than 2 g/kg, and the pH value was between 6.5 and 7.5.

**Table 11:** Preliminary prediction results of irrigation and nitrogen application range.

Days after sowing	Irrigating water quota (mm)		Nitrogen application rate (kg/hm^2^)	
	Upper limit	Lower limit	Upper limit	Upper limit
17d	102	93	71	65
59d	98	92	73	64
87d	98	92	81	72

### Evaluation and prospect of the PNN model

The superiority of PNN mainly depends on its unique “preference mechanism.” In general, data obtained from field trials based on human processing have high sampling and assay costs, so the data set obtained is often limited^37^. However, studies requiring control of specific variables must conduct field trials, thus the model must be in the limited data set for training in this field of study. Through the preferred connection between neurons, PNN can actively learn and adhere to the prior rules in the data so that PNN can achieve higher convergence and lower error rate than other models in the case of limited data than other machine learning models. In addition, existing artificial intelligence models or physical models are mostly used to predict monitoring indicators such as soil moisture and groundwater depth^38,39^, rather than artificial strategies like irrigation amount, but it should be an inevitable trend of intelligent agriculture that the model results directly guide production practice. Due to its reverse training structure, PNN can directly determine optimal irrigation and nitrogen application strategies based on multidimensional targets, which significantly improves the accuracy and versatility of the model. Benefiting from the above characteristics, the PNN model makes it possible to apply the deep learning model as a core decision-making tool in “precision agriculture”, and provides a solution for solving multidimensional decision-making problems in limited data sets.

## Conclusion

This study aims to predict the reasonable irrigation amount and N rate according to the multidimensional soil fertility objective. Therefore, a neural network model with 6-hidden layers and preference mechanism, namely the Preference Neural Network (PNN) model, has been developed. Four core indexes of soil fertility evaluation (organic matter, total nitrogen, total salt, pH), irrigation time and target soil depth were used as the input of PNN, and the amount of irrigation and nitrogen application were used as the output. The model was trained and tested using the data from 2018 to 2019, 80% of which was the training set, and 20% was the test set. The data from the validation test carried out in 2020 was used for verification. The results showed that the R^2^ values of PNN in predicting irrigation amount and N rate were 0.3356 to 0.7708 and 0.361 to 0.6721 higher than those of the models without neural network structure (support vector regression, linear regression and Logistic regression), respectively, and were 0.2609 and 0.2704 higher than the traditional neural network (BPNN) with one hidden layer, respectively. In addition, the R^2^ values of the predicted irrigation amount and N rate by PNN with the preference mechanism were 25.81% and 27.99% higher than those without the preference mechanism, respectively. It was proved that on the 17th, 59th and 87th day after seeding, the soil fertility could reach the standard of high grade with irrigation of 93 to 102, 92 to 98, 92 to 98 mm and nitrogen application of 65 to 71, 64 to 73 and 72 to 81 kg/hm^2^, respectively.

## Data Availability

Some data through which the mean, median, max, min and standard deviation were derived are available from the corresponding author upon reasonable request. Also surface figure through which models and forecasts were derived which support the findings of this study are available from the corresponding author.
